# Learning from Online Video Education (LOVE) improves confidence in fertility treatments: a randomized controlled trial

**DOI:** 10.1038/s41746-022-00673-y

**Published:** 2022-08-29

**Authors:** Amanda Adeleye, Katrina Cruz, Marcelle I. Cedars, Lauri Pasch, Heather Huddleston

**Affiliations:** 1 Section of Reproductive Endocrinology and Infertility, Department of Obstetrics and Gynecology, The University of Chicago 5841 South Maryland Avenue, MC 2050, Chicago, IL 60637 USA; 2 Center for Reproductive Health, Department of Obstetrics, Gynecology and Reproductive Sciences, University of California, San Francisco 499 Illinois St., San Francisco, CA 94158 USA

**Keywords:** Patient education, Randomized controlled trials

## Abstract

Fertility treatments like in vitro fertilization (IVF) or oocyte cryopreservation (OC) require the daily use of injectable gonadotropins and has been associated with treatment burden and attrition from fertility treatment. We conducted a randomized clinical trial to determine (1) whether educational videos about fertility medications improved infertility self-efficacy scale (ISES), fertility quality of life treatment (FertiQoL-T), and Perceived stress scale (PSS) scores and (2) if such videos improved confidence and reduced medication errors during a first ovarian stimulation cycle. Participants were given access to an online portal with randomized access to either placebo control videos focused on an orientation to IVF or experimental videos that reviewed the preparation and administration of medications used during ovarian stimulation in addition to the placebo videos. Participants completed pre and post-treatment questionnaires. 368 patients enrolled and 257 participants completed the study. There were no differences in ISES, FertiQoL-T or PSS scores between the two groups in an intention-to-treat (*p* = 0.18, 0.72, and 0.92, respectively) or per-protocol analysis (*p* = 0.11, 0.38, and 0.37, respectively). In the per protocol analysis, participants who watched experimental videos were four-fold more likely to report confidence administering medications OR 4.70 (95% CI: 2.10, 11.1; *p* < 0.01) and were 63% less likely to make medication errors OR 0.37 (95% CI: 0.14, 0.90; *p* = 0.03). Participants had similar likelihoods of rating videos as helpful and recommending videos to others (*p* = 0.06 and 0.3, respectively). Educational videos about fertility medications may not influence psychological well-being but might improve confidence in medication administration and reduce medication errors. Trial registration number: NCT02979990.

## Introduction

Many reproductive endocrinologists believe their counseling, along with nurse medication teaching, is sufficient to prepare patients for infertility treatments. Unfortunately, patients do not always feel prepared and may feel burdened by treatment. Treatment burden, which may be described as either the physical or psychological impacts of fertility treatment, is a significant reason for discontinuation of treatment even when patients have insurance coverage for infertility^1,2^. Additionally, studies have demonstrated a substantial proportion of patients forget medication instructions, lack self-confidence in administering their in vitro fertilization (IVF) medications and may not adhere to the instructions provided^3^. Addressing medication treatment burden may reduce treatment drop-out and possibly improve medication adherence, which is critical because the cumulative likelihood of pregnancy increases with each additional IVF cycle^4^.

One strategy to address treatment burden is to improve or innovate on the experience of learning about fertility medications. Instructional videos about medication usage have been useful in a variety of fields such as ophthalmology and pulmonology^5,6^. Within the infertility sector, mobile and computer-based technologies are emerging as another tool to address the patient experience with ovarian stimulation. While some interventions such as an online medication application, On Track, did not reduce medication errors in an randomized clinical trial, educational videos about the process of ovulation induction or IVF have demonstrably improved patient understanding of the process as well as the risks involved^7,8^. It is common practice for infertility centers to provide instruction on the use of IVF medications but in some cases, this information may be provided long before a patient actually initiates an ovarian stimulation cycle for fertility preservation or IVF. It is possible that instructional videos on fertility medications could reduce the medication treatment burden that infertility patients face by giving them easily accessible information about how to use IVF medications when they need it. To date, few studies have investigated whether educational videos about fertility medication usage could reduce treatment burden. For this reason, we developed the Learning from Online Video Education (LOVE) study, a randomized, double blinded, placebo-controlled clinical trial to explore whether educational videos about medications used for ovarian stimulation could positively impact patient well-being and adherence to the proper medication protocol.

Several validated questionnaires exist that at least partially capture the infertility patient treatment burden. The Fertility Quality of Life Treatment (FertiQoL-T) instrument has been used globally with a specific treatment questionnaire to evaluate treatment burden. Other instruments exist that partially characterize treatment burden and have also been associated with fertility treatment outcomes. In a pilot study from Turner et al, the Infertility Self Efficacy Scale (ISES), Perceived Stress Scale (PSS) and other psychological metrics, were measured in a group of 44 women undergoing their first IVF cycle. In this observational study, ISES and PSS scores collected prior to oocyte retrieval were associated with pregnancy outcomes even after adjusting for prognostic factors such as follicle count^9^. We hypothesized that LOVE study videos about ovarian stimulation medication preparation and administration could improve ISES scores. Further, we aimed to understand if study videos might influence secondary psychometric outcomes (FertiQoL-T and PSS scores). Finally we aimed to assess whether study videos improved self-reported confidence in medication administration, medication error rates and the need for provider assistance during a first ovarian stimulation cycle.

## Results

### Trial participants

**Fig. 1:**
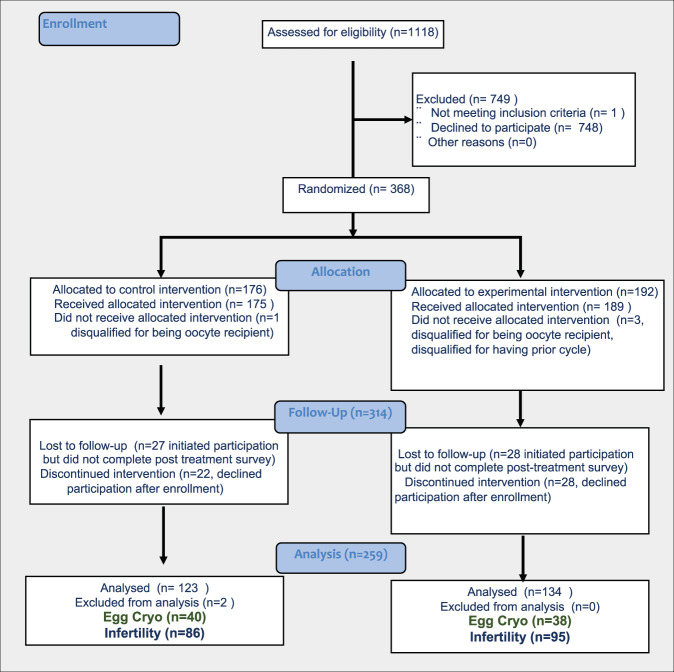
CONSORT flow diagram. Flowchart demonstrating the number of patients who were screened, enrolled, randomized and participated in the study.

1118 patients were screened for inclusion in the LOVE study. From these patients, 368 were enrolled; after randomization, 176 participants were allocated to the control group and 192 participants were allocated to the experimental group. After allocation, two participants were removed from the study for being oocyte recipients and two others were removed when it became known they had completed an ovarian stimulation cycle prior to enrollment. Among participants enrolled, 69.9% (*n* = 123) completed the final study survey in the control group and 69.8% (*n* = 134) completed the final survey in the experimental group. Two participants were excluded from analysis for being oocyte donors. Although oocyte donation was not an exclusion criterion at the outset, the psychometric tools used were not designed for an oocyte donor population (Fig. 1). Forty-nine participants in the control group and 56 participants in the experimental group enrolled in the study but did not complete the post-treatment survey. These participants were excluded from the final analysis. Participants who completed the study were slightly younger (36.38 +/− SD 3.79 years) than those who discontinued study participation or were lost to follow-up (37.17 +/− SD 4.39 years) (*p* = 0.04).

Demographic characteristics did not differ between control and experimental groups. The majority of participants identified as non-Hispanic White in both groups (65% and 60%, respectively). The mean age of participants was 35.9 in the control group and 36.5 in the experimental group. All participants had some college education or higher. In both groups, the majority of participants reported a household income of $150,000 or more (64% in the control group and 59% in the experimental group). There were no differences in participant age, race, education, or income between the control and experimental groups (*p* = 0.09, 0.6, 0.8, and 0.9, respectively) (Table 1). The demographics of the cohort are detailed in Table 1.

**Table 1:** Participant characteristics.

Characteristic	Control *N* = 123^a^	Experimental, *N* = 134^a^	*p*-value^b^
Age	35.9 (33.8, 38.5)	36.5 (34.6, 39.5)	0.09
Race			0.6
American Indian/Alaska Native	1 (0.8%)	0 (0%)	
Asian	30 (24%)	38 (28%)	
Black or African American	1 (0.8%)	4 (3.0%)	
White	80 (65%)	81 (60%)	
More than One Race	5 (4.1%)	3 (2.2%)	
Unknown/Not reported	6 (4.9%)	8 (6.0%)	
Education			0.8
High school	1 (0.8%)	0 (0%)	
Some college, trade school or associates	3 (2.5%)	5 (4.0%)	
Bachelor’s degree	37 (31%)	36 (29%)	
Some graduate school	6 (5.0%)	4 (3.2%)	
Graduate degree (masters, M.D., Ph.D, J.D.)	72 (61%)	79 (64%)	
Not reported	4	10	
Income			0.9
Less than $50,000	3 (2.5%)	4 (3.3%)	
$50,000 to $99,000	13 (11%)	14 (11%)	
$100,000 to %150,000	26 (22%)	32 (26%)	
$ Greater than $150,000	76 (64%)	73 (59%)	
Unknown	5	11	
Indication			0.6
Oocyte Cryopreservation	40 (33%)	38 (28%)	
IVF	83 (67%)	96 (72%)	
Watched study videos			0.9
No	49 (47%)	53 (47%)	
Yes	55 (53%)	59 (53%)	
Did not report	19	22	

^a^Statistics presented: median (IQR); *n* (%).

^b^Statistical tests performed: *t*-test; Fisher’s exact test; chi-square test of independence.

In the control group, 83 participants sought ovarian stimulation for IVF and 40 participants sought treatment for OC. In the experimental group, IVF was the indication for ovarian stimulation for 96 of the participants and OC for 38 participants. There was no difference in the indication for treatment between control and experimental groups (*p* = 0.6) (Table 1).

### Intervention

To be included in analysis, participants must have completed the ISES survey, however, they were not required to answer other survey questions nor were they required to watch study videos. Forty-one participants did not answer whether or not they watched the study videos, although the majority of their survey responses were completed and included in the intention-to-treat analysis. Of the 216 participants who answered whether or not they watched the study videos, 52.9% (*n* = 55) of participants in the control group and 52.7% (*n* = 59) of participants in the experimental group reported watching the study videos. There was no difference in uptake of study intervention between the two groups *p* = 0.98 (Table 1).

### Infertility self-efficacy

For the primary outcome of ISES, the mean ISES score for the control group was 99.27 +/− (21.41) and the mean ISES score for the experimental group was 99.08 +/− (23.54). In an intention-to-treat analysis, there was no difference in ISES scores between the two groups (*p* = 0.95). Among exclusively infertile participants, the mean ISES score for the control group (*n* = 83) was 100.07 +/− (20.52) and the mean ISES score for the experimental group (*n* = 96) was 95.22 +/− (22.58). There were no differences in ISES scores between the two groups using an intention to treat analysis (*p* = 0.18) (Table 2). In a per-protocol analysis of participants treated with IVF, there was no difference in ISES scores between IVF participants in the control and experimental groups (*p* = 0.11) (Table 3).

**Table 2:** Outcomes from intention to treat analysis.

	Control	Experimental	*P* Value
ISES	99.27 +/− 21.41	99.08 +/− 23.54	0.95
ISES^a^	100.07 +/− 20.52	95.22 +/− 22.58	0.18
FertiQoL-T	27.3 +/− 5.48	27.04 +/− 5.25	0.72
PSS	16.9 +/− 6.8	16.82 +/− 5.77	0.92

^a^Intention to treat for infertility patients only.

**Table 3:** Outcomes from per protocol analysis.

	Control (*n* = 55)	Experimental (*n* = 59)	*P* Value
ISES	99.62 +/− 39.00	98.23 +/− 46.53	0.11
FertiQoL-T	26.78 +/− 11.17	27.48 +/− 9.62	0.48
PSS	16.49 +/− 12.76	17.54 +/− 11.44	0.37
Patient video experience			
Found videos helpful	76.4% (*n* = 42)	89.8% (*n* = 53)	0.06
Felt confident taking medication	45.5% (*n* = 25)	79.7% (*n* = 47)	<0.01
Made a medication error	32.7% (*n* = 18)	15.3% (*n* = 9)	0.03
Would recommend videos to others	81.8% (*n* = 45)	88.1% (*n* = 52)	0.30
Sought assistance for medication questions	32.7% (*n* = 18)	20.3% (*n* = 12)	0.14

### Fertility quality of life

The mean FertiQoL-T score for the control group was 27.3 +/− (5.48) compared with 27.04 +/− (5.25) for the experimental group. There was no difference in FertiQoL-T scores between the two groups using an intention to treat analysis for the entire LOVE study cohort (*p* = 0.72) or in a per protocol analysis (*p* = 0.48) (Tables 2, 3). There was no difference between the two groups when assessed by indication for treatment (IVF vs. OC) (*p* = 0.86, or 0.14 respectively).

### Perceived stress scale

The mean PSS score for the control and experimental groups were 16.9 +/− (6.8) and 16.8 +/− (5.77), respectively. There were no differences in PSS scores collected before and after treatment for each group (*p* = 0.94 and 0.25, respectively). For additional analysis comparing pre-treatment and post-treatment PSS scores in the LOVE study cohort, readers may refer to our previously published study on this topic^10^. There was no difference in PSS score between the two groups using an intention to treat analysis for the entire LOVE study cohort (*p* = 0.92) (Table 2). In a per protocol analysis of the entire cohort there was no difference in the PSS score between the two groups (*p* = 0.37) (Table 3).

When stratifying by the indication for treatment (IVF vs. OC), in an intention to treat analysis, there were no differences in PSS scores by exposure group (*p* = 0.45 and 0.12, respectively). In a per protocol analysis, when stratifying by the indication for treatment, there was no difference in PSS post-treatment scores for OC participants (*p* = 0.12). IVF participants in the experimental group had higher post-treatment PSS scores (18.37 +/− 5.93) than participants in the control group (15.9 +/− 6.39) and this neared significance (*p* = 0.07).

### Patient experience

In a per-protocol analysis, participants who watched the experimental videos endorsed more confidence in taking injectable medications compared to participants in the control group using a univariate logistic regression OR 4.70 (95% CI: 2.10, 11.1; *p* < 0.001). Participants who watched the experimental study videos were also less likely to report making medication administration errors OR 0.37 (95% CI: 0.14, 0.90; *p* = 0.031). Experimental group participants found their videos to be more helpful relative to participants in the control group; this difference neared, but did not meet, statistical significance OR 2.73 (95% CI: 0.99, 8.35; *p* = 0.06). There was no difference in a participant’s likelihood to recommend videos to others going through ovarian stimulation OR 1.65 (95% CI: 0.59, 4.89; *p* = 0.3). Although participants in the experimental group found the videos to be helpful and reduced the rate of medication errors, it did not change the likelihood for participants to report asking for assistance with medications from medical staff OR 0.52 (95% CI: 0.22, 1.21; *p* = 0.14) (Table 3).

## Discussion

Although infertility clinics may provide comprehensive counseling on fertility treatments, some patients will have difficulties adhering to their medication regimen. The LOVE study demonstrated that instructional videos improved patient confidence in a first ovarian stimulation cycle and reduced self-reported medication errors. The importance of these outcomes cannot be overstated, as interventions that can alleviate the significant treatment burden of IVF may aid in the reduction of treatment drop-out and improve chances for patients to reach their ultimate goal of becoming parents^4^.

Within the field of infertility, the LOVE study is one of the first studies to demonstrate that some important aspects of the treatment experience can be improved with educational interventions. Other studies, such as a randomized clinical trial about a writing intervention for infertile couples have demonstrated that treatment burden could be improved, but few studies have looked specifically at how technology such as instructional videos can accomplish this goal^11^. The medication error rate for the placebo group in the LOVE study was similar to other studies. However, patients who utilized experimental instructional study videos during their first cycles had a significant reduction in medication errors.

We did not observe a difference in our measures of self-efficacy, stress or treatment burden (quantified through FertiQol-T) among participants assigned to experimental videos compared to control videos. There are some aspects of our protocol, particularly for the control group that may have influenced our results. All patients, independent of study assignment were required to attend an in-person IVF medication teaching course. Additionally, control participants had access to placebo control videos. The intent of the placebo control videos was to help distinguish the effect of having access to any videos to the impact of accessing educational content. Because all participants had access to general videos about the ovarian stimulation process, it allowed participants to be blinded to their study group without introducing video content that would be unique to the control group.

This fairly robust placebo control protocol may have led to efficacy and stress benefits for control participants, making it more difficult to detect a difference relative to the intervention group. All participants were required to attend an IVF medication class which may have built a sufficient fund of knowledge for participants thus making interventional videos less helpful or necessary. Furthermore, we did not assess whether participants suspected their group allocation. It is conceivable that unintentional unblinding by participants could have influenced the study outcomes.

However, this was a large, well-controlled study that enrolled a racially and socio-economically diverse population. The LOVE study sought to closely emulate real-world settings by not limiting participants ability access educational resources. Additionally, this study included both patients with infertility and those seeking planned oocyte cryopreservation which may widen the applicability of the results.

There were several limitations to this study. One constraint was in identifying appropriate metrics to assess the impact of the experimental videos on the study population. Our most direct questions about confidence and medication errors were not previously validated. In contrast, our validated metrics, ISES, FertiQoL-T and PSS were excellent descriptors of self-efficacy and well-being, but did not comprehensively capture the aspects of treatment burden we hypothesized might be affected by experimental study videos. Furthermore, metrics such as anxiety were not discretely assessed. Fortunately, previous studies have demonstrated that FertiQoL is associated with standardized measures of anxiety and we hope, at least partially captured elements of anxiety among LOVE study participants^12^. Additionally, our study population included women who were interested in planned oocyte cryopreservation, however our primary outcome, the infertility self-efficacy scale, was not intended for use in an OC population. Restricting our analysis to infertility patients exclusively did not change our findings.

There were demographic factors such as prognosis and medical history that were not accounted for in this study. It is possible that participants may have assessed their prognosis during treatment, even prior to egg retrieval. In such cases it is possible that these participants may have scored lower on our outcomes of interest. Furthermore, we did not explicitly evaluate the reading level of the survey or study intervention; this could have limited the ability to ascertain a difference between study groups. Fortunately, the validated questionnaires have been tested in infertility populations similar to our patient population and the education level between groups did not differ. Prior knowledge of medication administration could have influenced our results. All patients participated in an in-person IVF medication course. This knowledge alone could have supported enough patients such that the experimental study videos were superfluous. In this case, the experimental study videos would have limited effect. Furthermore we did not screen patients to determine whether or not they had previous experience with medications administered subcutaneously (i.e., insulin); if they had a medical background or worked in healthcare all of which could have influenced their comfort with medication administration at the outset. For these participants, the study videos may not have influenced their treatment burden. Our randomization strategy should have protected against biases from these demographic confounders. Measurable demographics did not differ between the control and experimental groups.

Although enrollment was robust, the dropout rate of 30.2% was higher than expected and as a result, the study did not have the power to find the differences delineated at the outset of the study. Fortunately, enough participants utilized the study videos to make an assessment about the participant centered value of instructional videos.

Ultimately, although clinic-developed videos on medication administration may not lead to an impact on infertility self-efficacy or perceived stress, we found that they did improve patient-reported confidence in medication administration and resulted in a reduction in medication errors during a first cycle of treatment, suggesting that other clinics may consider developing their own videos to aid in the reduction of treatment burden.

## Methods

### Trial oversight

The LOVE study was a randomized double-blinded placebo-controlled trial conducted between February 1st, 2017 and March 30th, 2018 at an academic medical center. This study was registered as a clinical trial (NCT02979990). The LOVE study received Institutional Review Board approval from the University of California San Francisco (IRB 16–20821). All participants provided written consent to take part in the LOVE study.

### Participants

All patients undergoing their first ovarian stimulation cycle for either planned oocyte cryopreservation (OC) or IVF at our medical center were recruited for participation in the study. To be included in the study, patients were required to be over the age of 18 and to have internet access. In our practice, all patients, regardless of study enrollment, were required to participate in a two hour IVF medication course. This course was taught by one of our nurse practitioners who would review the preparation and administration of the most common IVF medications. Participants would typically participate in the class shortly after they had confirmed a plan for ovarian stimulation with their doctor. However, the medication course may have been remote from when they actually initiated ovarian stimulation weeks to months later. On rare occasion, a patient may have deferred the IVF medication class in which case the patient would be excluded from enrolling in the LOVE study.

Patients were also excluded if their physician advised against participation the study, or if a patient had previously completed an ovarian stimulation cycle prior to enrollment.

### Development of the study intervention

LOVE study videos were developed by a multidisciplinary team of physicians and nurses. There were two types of LOVE study videos: placebo control videos, which will be referred to as “control videos” and experimental videos. Control videos included a video previously developed by our clinic that served as an orientation to IVF that was available to all patients regardless of enrollment status in the LOVE study. Participants in the control group also had access to a video about the physiology of ovarian stimulation developed by the LOVE study team. Experimental videos reviewed common medications that participants might encounter during treatment. Each video included an explanation of what supplies were needed for medication administration and a demonstration of how to administer the medication. Experimental video topics included: information about needles, preparation of the space for medication administration, a follicle stimulating hormone (FSH) agent, a human menopausal gonadotropin (hMG) agent, the human chorionic gonadotropin (hCG) “Trigger shot”, progesterone preparation, and progesterone injection. Study videos are available for public access at https://crh.ucsf.edu/medication-videos

**Fig. 2:**
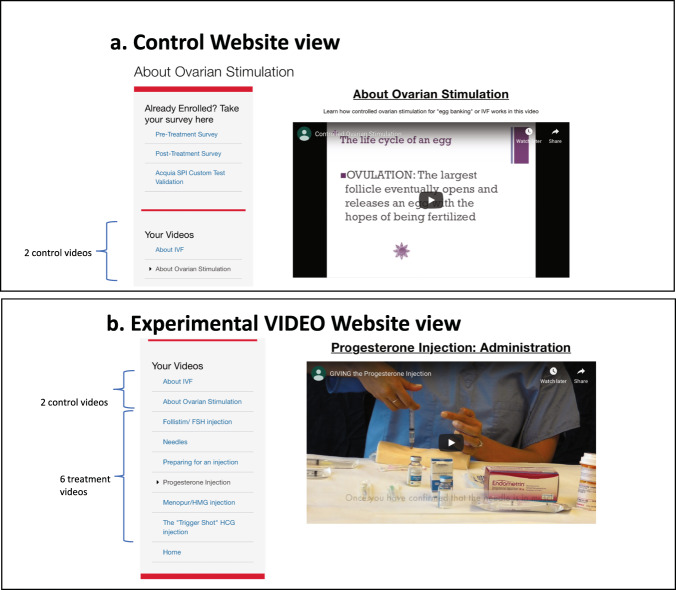
LOVE study website. Screenshots of the online portal for the (**a**) control group and (**b**) experimental group.

Each participant was given a unique username and password to the study website (Fig. 2). Once logged in, their account would provide them with access to videos depending on their treatment group. Control group participants had access to control videos only, whereas experimental group participants had access to control videos and experimental videos to protect against the possibility of an independent effect of the placebo videos.

### Trial procedures

As per our inclusion criteria, all participants attended our in-person, IVF medication teaching course. Patients who enrolled in the LOVE study were randomized in a 1:1 ratio to a control or experimental group. Randomization was accomplished by assigning either control or experimental access to videos on the study platform for each study account. The account randomization was computerized and accounts were assigned by a research associate who did not participate in recruitment or analysis of the study. Participants were informed that study videos were educational but the precise content of the videos was not described. Both participants and investigators were blinded to the patient study assignment. Participants were not required to watch study videos. However, participants in the control group who chose to log in to the study website had access to “control” videos on the process of ovarian stimulation and an IVF orientation. Participants in the experimental group had access to all videos: the “experimental” videos on IVF medications and also control videos. Unmasking of study assignments occurred after study closure at the time of initial data analysis. Study closure occurred after target enrollment was reached however the indication for ovarian stimulation for participants was not assessed.

Participants were asked to complete surveys online, administered through Redcap at the time of enrollment and at the end of ovarian stimulation^13^. The initial survey included questions about demographic data and a 10-item PSS, completed prior to the start of their ovarian stimulation cycle and up to the second day of ovarian stimulation. The second survey was completed at the time of trigger for the final maturation of oocytes up to 16 days after the trigger injection and prior to a determination of pregnancy status. Patients who completed the final survey after this time point were excluded from analysis.

Importantly, participants were encouraged but not required to watch study videos. Furthermore, other instructional videos that were not produced by the LOVE study were routinely available to patients. Participants were neither encouraged nor discouraged from accessing additional educational content. This scenario most closely represented the educational environment typical patients would encounter in our clinical setting.

### Outcomes

The primary outcome of the LOVE study was the ISES score at the end of ovarian stimulation. Although ISES was developed to describe infertility patients’ perceptions about diagnosis and treatment more broadly, given its potential to be associated with clinical differences we employed it in this study as a measure of treatment burden wherein higher ISES scores may represent less treatment burden. The ISES score is derived from a 16-item questionnaire scored from 1 to 9 on a Likert scale. Scores could range from 16 to 144. Higher scores were associated with a stronger self-assessment of one’s ability to cope with an infertility diagnosis and treatment. In a prospective cohort study of 44 women, ISES scores were approximately 10% higher at the time of egg retrieval in women who conceived in that cycle. We deemed a difference of 10% in ISES scores as clinically important, with the potential to have both psychological and treatment-related benefits.

There were multiple secondary outcomes. We employed the Ferti-QoL-T questionnaire which is used internationally to assess the general and treatment related quality of life for people experiencing infertility. The treatment portion of the instrument consists of 10 items that gauge patients’ quality of life as it relates to fertility treatment and is scored from 0 to 4. Higher scores are associated with a higher quality of life with a maximum score of 40^14^.

We also utilized the Perceived Stress Scale (PSS), which was first developed by Sheldon Cohen et al.,^15,16^. The PSS has been used in a wide variety of settings and scores are well correlated with other measurements of stress^10,17^. The PSS score is based upon a 10-item questionnaire to assess stress in the last month. Scores could range between 0 and 40 with higher scores being associated with higher levels of stress^16^. Finally, participants were queried as to whether or not they felt confident about taking assigned medications and if they found the videos helpful, made any medication errors, required medication assistance from staff, or would recommend the videos to others. Participants were also queried as to whether or not they watched the videos.

### Statistics

We hypothesized that participants exposed to the experimental videos would have higher ISES scores than patients exposed to control videos. For the primary outcome of ISES scores, 107 participants would be needed in each group to detect a 10% difference at a power of 0.8 with a standard deviation of 24.5. Target enrollment for this study was 250 participants seeking IVF assuming a dropout rate of 14% which was similar to other infertility-related randomized clinical trials^18,19^.

Demographic data between the control and experimental groups were assessed using *t*-tests or chi-squared tests where appropriate. Continuous outcomes (ISES, FertiQoL-T and PSS scores) were compared between experimental and control groups using a two-sided *t*-test in an intention-to-treat and per-protocol analysis which restricted analysis to patients that endorsed watching study videos. Participant assessments of the video quality – whether or not they found videos to be beneficial (helpful, improving confidence, worthy of recommending etc.) was assessed using univariate logistic regressions.

Normality of the primary outcome (ISES score) was confirmed with the Shapiro-Wilk test. Data are presented as point estimates and significance was determined at *p* < 0.05. The data were analyzed using R version 3.6.3.

### Reporting summary

Further information on research design is available in the Nature Research Reporting Summary linked to this article.

## Supplementary information

Reporting Summary

## Data Availability

Public data sharing was not an element approved by the UCSF IRB at the time of study initiation. Questions regarding a minimal dataset should be directed to the corresponding author. Qualified researchers can apply for access to the datasets by contacting the corresponding author.
