# Supplementary material for: Learning from Online Video Education (LOVE) improves confidence in fertility treatments: a randomized controlled trial
Source: NPJ Digit Med. 2022 Aug 29;5:128. doi: 10.1038/s41746-022-00673-y (PMC9424217; doi:10.1038/s41746-022-00673-y)
Supplement: Supplementary file 1 — Reporting Summary [file 41746_2022_673_MOESM1_ESM.pdf]

nature portfolio

Corresponding author(s): Amanda Adeleye
DBPR NPJDIGITALMED-03376

Last updated by author(s): May 25, 2022

Reporting Summary

Nature Portfolio wishes to improve the reproducibility of the work that we publish. This form provides structure for consistency and transparency in reporting. For further information on Nature Portfolio policies, see our Editorial Policies and the Editorial Policy Checklist.

Statistics

For all statistical analyses, confirm that the following items are present in the figure legend, table legend, main text, or Methods section.

n/a	Confirmed	
☐	☒	The exact sample size (n) for each experimental group/condition, given as a discrete number and unit of measurement
☐	☒	A statement on whether measurements were taken from distinct samples or whether the same sample was measured repeatedly
☐	☒	The statistical test(s) used AND whether they are one- or two-sided<br>*Only common tests should be described solely by name; describe more complex techniques in the Methods section.*
☐	☒	A description of all covariates tested
☐	☒	A description of any assumptions or corrections, such as tests of normality and adjustment for multiple comparisons
☐	☒	A full description of the statistical parameters including central tendency (e.g. means) or other basic estimates (e.g. regression coefficient) AND variation (e.g. standard deviation) or associated estimates of uncertainty (e.g. confidence intervals)
☐	☒	For null hypothesis testing, the test statistic (e.g. F, t, r) with confidence intervals, effect sizes, degrees of freedom and P value noted<br>*Give P values as exact values whenever suitable.*
☒	☐	For Bayesian analysis, information on the choice of priors and Markov chain Monte Carlo settings
☒	☐	For hierarchical and complex designs, identification of the appropriate level for tests and full reporting of outcomes
☒	☐	Estimates of effect sizes (e.g. Cohen's d, Pearson's r), indicating how they were calculated

Our web collection on statistics for biologists contains articles on many of the points above.

Software and code

Policy information about availability of computer code

Data collection	Survey data were collected using Redcap
Data analysis	R version 3.6.3

For manuscripts utilizing custom algorithms or software that are central to the research but not yet described in published literature, software must be made available to editors and reviewers. We strongly encourage code deposition in a community repository (e.g. GitHub). See the Nature Portfolio guidelines for submitting code & software for further information.

Data

Policy information about availability of data

All manuscripts must include a data availability statement. This statement should provide the following information, where applicable:

- Accession codes, unique identifiers, or web links for publicly available datasets
- A description of any restrictions on data availability
- For clinical datasets or third party data, please ensure that the statement adheres to our policy

Public data sharing was not an element approved by the UCSF IRB at the time of study initiation. Questions regarding a minimal dataset should be directed to the corresponding author. Qualified researches can apply for access to the datasets.

Field-specific reporting

Please select the one below that is the best fit for your research. If you are not sure, read the appropriate sections before making your selection.

☒ Life sciences ☐ Behavioural & social sciences ☐ Ecological, evolutionary & environmental sciences

For a reference copy of the document with all sections, see nature.com/documents/nr-reporting-summary-flat.pdf

Life sciences study design

All studies must disclose on these points even when the disclosure is negative.

Sample size	We hypothesized that participants exposed to the experimental videos would have higher ISES scores than patients exposed to placebo videos. For the primary outcome of ISES scores, 107 participants would be needed in each group to detect a 10% difference at a power of 0.8 with a standard deviation of 24.5. Target enrollment for this study was 250 participants with infertility assuming a dropout rate of 14 % which was similar to other infertility related randomized clinical trials<br>368 were enrolled; after randomization, 176 participants were allocated to the control group and 192 participants were allocated to the experimental group. In the control group, 83 participants sought ovarian stimulation for infertility and 40 participants sought treatment for oocyte cryopreservation. In the experimental group, infertility was the indication for treatment for 96 of the participants and oocyte cryopreservation for 38 participants.
Data exclusions	Two participants were excluded from final analysis for being oocyte donors. This characteristic was not considered during the design of the study as an exclusion criteria but the metrics used were not designed for this situation.
Replication	There were no attempts made to replicated the findings of this study
Randomization	Patients who enrolled in the LOVE study were randomized in a 1:1 ratio to a control or experimental group. Randomization was accomplished by assigning either control or experimental access to videos on the study platform for each study account. The account randomization was computerized using the excel random function to assign accounts to study groups. and accounts were assigned by a research associate who did not participate in recruitment or analysis of the study.
Blinding	Investigators and participants were blinded to participant study allocation which was determined by the study account type assigned to each patient.

Reporting for specific materials, systems and methods

We require information from authors about some types of materials, experimental systems and methods used in many studies. Here, indicate whether each material, system or method listed is relevant to your study. If you are not sure if a list item applies to your research, read the appropriate section before selecting a response.

Materials & experimental systems

n/a	Involved in the study	
☒	☐	Antibodies
☒	☐	Eukaryotic cell lines
☒	☐	Palaeontology and archaeology
☒	☐	Animals and other organisms
☐	☒	Human research participants
☐	☒	Clinical data
☒	☐	Dual use research of concern

Methods

n/a	Involved in the study	
☒	☐	ChIP-seq
☒	☐	Flow cytometry
☒	☐	MRI-based neuroimaging

Human research participants

Policy information about studies involving human research participants

Population characteristics	Reproductive aged women aged 18 years and older who were interested in ovarian stimulation for either oocyte cryopreservation (egg banking) or in vitro fertilization (IVF). Women seeking IVF had infertility or were attempting to avoid transmitting a genetically inherited condition to their offspring using preimplantation genetic testing for aneuploidy. All women lived in the Bay area in the United States and received care at the University of California San Francisco Center for Reproductive Health
Recruitment	Eligible participants attended an IVF medication teaching session in person that demonstrated the preparation and use of the most common medications for cycles in our clinic. A study investigator gave a brief presentation during the medication class reviewing the general elements of the study and was available to enroll patients at the end of the class
Ethics oversight	University of California Internal Review Board

Note that full information on the approval of the study protocol must also be provided in the manuscript.

Clinical data

Policy information about clinical studies

All manuscripts should comply with the ICMJE guidelines for publication of clinical research and a completed CONSORT checklist must be included with all submissions.

Clinical trial registration	NCT02979990
Study protocol	The study protocol can be accessed at clinicaltrials.gov. https://clinicaltrials.gov/ct2/show/NCT02979990
Data collection	Setting Academic medical center - reproductive endocrinology and infertility practice, locale San Francisco, CA, USA, time period 2/1/2017 -2018
Outcomes	In a pilot study from Turner et al, the Infertility Self Efficacy Scale (ISES), Perceived Stress Scale (PSS) and other psychological metrics, were measured in a group of 44 women undergoing their first IVF cycle. In this observational study, ISES and PSS scores collected prior to oocyte retrieval were associated with pregnancy outcomes even after adjusting for prognostic factors such as follicle count (Turner et al., 2013). We attempted to determine if our intervention could lead to a difference in ISES scores of 10% which would approximate conditions in the Turner study which were associated with differing pregnancy rates. Secondary outcomes were chosen for their hypothesized relevance to patient treatment burden.
